# Normative data and clinical usability of the brief examination of social abilities: a novel screening test for social cognition

**DOI:** 10.1007/s10072-026-09173-3

**Published:** 2026-06-15

**Authors:** Maria Franca, Chiara Gramegna, Desirè Carioti, Caroline Levi Morenos, Gaia Sangalli, Laura Perucca, Stefano Zago, Nadia Bolognini

**Affiliations:** 1 Department of Medicine & Surgery, PhD Program in Neuroscience, University of Milano-Bicocca, Monza, Italy; 2 Department of Psychology, University of Milano-Bicocca, Milan, Italy; 3 Department of Psychiatry, Psychotherapy and Psychosomatics, Faculty of Medicine, RWTH, Aachen, Germany; 4 Laboratory of Neuropsychology, Department of Neurorehabilitation Sciences, IRCCS Istituto Auxologico Italiano, Milan, Italy; 5 Department of Neurorehabilitation Sciences, IRCCS Istituto Auxologico Italiano, Milan, Italy; 6 Department of Biomedical Sciences For Health, University of Milan, Milan, Italy; 7 Fondazione IRCCS Ca’ Granda, Ospedale Policlinico, Neurology Unit, Milan, Italy

**Keywords:** Social cognition, Neuropsychological assessment, Screening test, Normative data

## Abstract

**Background:**

Social cognition impairments are common across neurological and psychiatric conditions and are associated with poor functional outcomes, yet their systematic assessment in routine clinical practice remains limited. This study aimed to introduce a novel screening battery for social cognition, the Brief Examination of Social Abilities (BE-Social), to provide Italian normative data and evidence on its clinical usability.

**Methods:**

The BE-Social assesses various socio-cognitive abilities (i.e., social perception, Theory of Mind (ToM), empathy, and social norm understanding) through seven tasks administered in approximately 15 min. Normative data were collected from 466 healthy adults, and clinical usability was assessed in 76 patients with neurological and psychiatric disorders. Psychometric properties, including internal consistency, test–retest reliability, and construct validity, were examined. Normative adjustments were derived using the Equivalent Score method. Receiver operating characteristic (ROC) analyses were performed to evaluate discriminative accuracy.

**Results:**

The BE-Social shows satisfactory internal consistency (McDonald’s ω = 0.83) and moderate test–retest reliability (ICC = 0.66). Its score is associated with standardized measures of emotion recognition and ToM, but not with executive functions, attention, or memory, supporting its specificity. Age and education, but not sex, significantly predict performance. The BE-Social demonstrates good accuracy in distinguishing socio-cognitively impaired from non-impaired patients (AUC = 0.82). In patients, BE-Social scores are associated with emotion recognition, ToM, and social norm understanding, with these associations remaining significant after controlling for global cognitive functioning.

**Conclusions:**

The BE-Social is a brief, reliable, and clinically feasible screening tool for assessing social cognition, supporting its use in routine assessment across neurological and psychiatric populations.

**Supplementary Information:**

The online version contains supplementary material available at 10.1007/s10072-026-09173-3.

## Introduction

Social cognition refers to a broad set of cognitive and affective processes that enable individuals to perceive, interpret, and respond appropriately to social information, supporting effective interpersonal interactions and social integration [1]. Although its internal structure remains debated, it is consistently described as comprising a core set of domains, namely social perception, Theory of Mind (ToM), empathy, and social and moral norm understanding [2].

Together, these abilities allow individuals to infer others’ mental states, regulate social behavior, and navigate complex interpersonal contexts.

Converging evidence has demonstrated that impairments in social cognition are not confined to specific diagnostic categories but occur across a wide range of neurological and psychiatric conditions [2–4]. These deficits are associated with clinically meaningful outcomes, including difficulties in maintaining interpersonal relationships, reduced occupational functioning, and poorer community integration [5, 6]. Consistent with this view, the Diagnostic and Statistical Manual of Mental Disorders (DSM-5) [7] included social cognition among the six core neurocognitive domains, acknowledging its relevance across neurologic, neuropsychiatric, and neurodevelopmental disorders. Within this framework, social cognition has increasingly been conceptualized as a transdiagnostic domain, whose assessment may provide valuable information across diagnostic boundaries [2, 3, 6, 8].

Importantly, the clinical expression and severity of socio-cognitive impairment can vary considerably across individuals and clinical conditions. These difficulties may also change over time within the same condition, for example, as a function of disease stage or progression [4]. For these reasons, the accurate identification of social cognition impairments is relevant not only for research purposes but also for clinical formulation, treatment planning, and monitoring.

Importantly, reduced awareness of socio-cognitive deficits (i.e., anosognosia) may further complicate their detection and treatment, as patients may underestimate or fail to recognize their own social difficulties. Anosognosia may be domain-specific, with patients showing impaired insight into social cognition (e.g., ToM impairments) despite preserved awareness of other cognitive deficits [9].

Despite its recognized relevance, the systematic assessment of socio-cognitive abilities in routine clinical practice remains limited [6, 10–12]. International surveys and position papers consistently show that social cognition is rarely evaluated using standardized tools, even in populations where impairments are well documented. Reported barriers include limited availability of validated instruments, lack of normative data, uncertain psychometric properties, time constraints, and insufficient training in social cognition assessment [10–12]. Similar trends have been documented in Italy, where the use of standardized social cognition tests remains extremely low in neurological and geriatric settings [13].

Although numerous tasks exist to assess specific socio-cognitive abilities, most focus on a single domain or require lengthy administration. Only a few batteries, such as the Mini-Social and Emotional Assessment [mini-SEA; 14] or the short version of The Awareness of Social Inference Test [TASIT-S; 15], evaluate more than one component within a relatively brief protocol. However, they typically focus on emotion recognition and ToM, while providing more limited coverage of other clinically relevant aspects such as empathy or social norm understanding.

In the Italian context, brief and multidomain batteries supported by appropriate normative data remain extremely limited, substantially constraining the integration of social-cognition assessment into routine neuropsychological practice.

One of the few available instruments is the Italian battery proposed by Prior et al. [16], which assesses multiple aspects of social cognition, including ToM, emotion attribution, social judgments, as well as moral–conventional distinctions.

The present study aims to introduce a novel screening test for social cognition, the Brief Examination of Social Abilities (BE-Social), and to examine its psychometric properties; provide normative data for the Italian population and offer preliminary evidence on its clinical usability in neurologic and psychiatric patients.

## Materials and methods

### Participants

The normative sample consisted of 466 Italian healthy adults (HC), recruited through institutional networks and community-based outreach. Exclusion criteria included: history of neurological or psychiatric disorders, severe or uncompensated general medical conditions, uncorrected auditory or visual impairments that could interfere with task performance, and a Montreal Cognitive Assessment (MoCA) adjusted score below the national cut-off (i.e., 18.58) [17]. Sample stratification by age, education, and sex is reported in Table 1.

**Table 1:** Stratification of the normative sample by age, education, and sex

			Education (years)			
	≤ 8	9–13	14–18	> 18	Total (F/M)	Total N
**20–29**	0/1	25/19	55/15	2/3	82/38	120
**30–39**	0/1	6/8	4/6	3/2	13/17	30
**40–49**	4/0	9/6	6/5	4/2	23/13	36
**50–59**	8/10	40/34	24/11	5/1	77/56	133
**60–69**	7/7	15/19	9/7	1/2	32/35	67
**70–79**	15/4	8/4	4/4	1/1	28/13	41
**>80**	17/6	8/5	2/1	0/0	27/12	39
**Total (F/M)**	51/29	111/95	104/49	16/11	286/184	466
**Total N**	80	206	153	27	466	-

*F *female, *M* male. Values represent the number of participants reported as female/male in each cell

The clinical sample consisted of 76 patients with neurological or psychiatric disorders. Neurological patients were recruited at the Istituto Auxologico Italiano (Capitanio Hospital, Milan), while psychiatric patients were recruited at the Fondazione IRCCS San Gerardo (Monza). Neurological patients (*n* = 42) presented traumatic brain injury (*n* = 7), vascular lesions (*n* = 11; i.e., ischemic or haemorrhagic stroke, aneurysm rupture, and arteriovenous malformations), focal non-traumatic lesions (*n *= 8; i.e., brain tumours and cerebellar abscess), as well as patients with neurodegenerative diseases (Parkinson’s disease: *n* = 13; frontotemporal dementia: *n* = 3). Psychiatric patients (*n* = 34) were diagnosed with schizophrenia spectrum disorders (*n* = 22), schizoaffective disorder (*n* = 7), and other non-affective psychotic disorders (*n* = 5). Patients were excluded if they had: current or past neurological or psychiatric diagnoses other than the condition of interest, severe comprehension deficits, uncorrected visual impairments, or other medical conditions that could impede full participation in the assessment.

All procedures were conducted in accordance with the Declaration of Helsinki, and the study was approved by the Ethics Committees of the University Milano-Bicocca (RM-2023–721) and Istituto Auxologico Italiano (N° 25C122). Written informed consent was obtained from all participants before inclusion in the study.

### The BE-Social

The BE-Social is designed to assess multiple processes of social cognition within a concise and clinically feasible protocol that can be administered at the bedside or in routine neuropsychological settings. The selection of the investigated abilities was informed by converging evidence from the social cognition literature, including clinical reviews and theoretical frameworks, which consistently identified these skills as core components of social cognition and as frequent targets of impairment across neurological and psychiatric conditions [2, 5]. Specifically, it consists of seven tasks, each targeting a specific socio-cognitive process. Task construction was grounded in established neuropsychological paradigms widely used in the literature, while original stimuli were developed when necessary to ensure novelty and avoid overlap with existing instruments. Its development was preceded by a pilot study aimed at refining task structure and stimulus selection; details of the pilot phase are reported in the Supplementary Materials S1.

*Social perception* was assessed through two tasks targeting identity recognition and basic emotion recognition:

- *The Identity Recognition task (IRt)* evaluates perceptual face discrimination without memory demands and was inspired by classical face-matching paradigms, such as the Benton Facial Recognition Test [18]. Participants are required to match a target face to one of three alternatives presented simultaneously, differing in viewpoint and lighting conditions. The target stimulus and the alternatives remained visible until the participant responded. The task includes 3 trials (score range: 0–3), scored dichotomously (correct = 1; incorrect = 0).
- *The Emotion Recognition task (ERt)* assesses the ability to identify basic facial emotions and was developed following paradigms commonly used in facial emotion recognition tasks, such as the Ekman 60 Faces Test [19]. Participants are shown faces expressing one of six basic emotions (anger, disgust, sadness, surprise, happiness, and fear) and are asked to select the corresponding emotional label. The task includes 6 trials (score range: 0–6), scored as correct or incorrect.

*ToM* was assessed using two tasks varying in modality and inferential demands, to capture different levels of ToM complexity:

- *The Story Inference task (SIt)* is a non-verbal task designed to assess first-order ToM and was inspired by the Story-based Empathy Task [20]. Participants view short sequential photographic vignettes depicting everyday situations without verbal input. Three conditions are included: causal inference (control condition), emotion inference (affective ToM), and intention inference (cognitive ToM). After observing each story, participants select the most appropriate ending from three alternatives. The task includes 3 trials (score range: 0–3), scored as correct or incorrect.
- *The Mini Faux Pas task (MFPt)* assesses second-order ToM through the detection and interpretation of social faux pas. The task was adapted from the Faux Pas Test originally developed by Stone et al. [21] and from its Italian adaptation for adults. Short stories are read aloud by the examiner. Two preliminary control questions are used to ensure adequate comprehension of the story and explicit recognition of the faux pas; only if these are answered correctly, participants proceed to the subsequent ToM questions, which assess the speaker’s intentions and the emotional impact on the recipient. Only ToM-related questions contribute to the final score. The task includes 2 trials (score range: 0–6).

*Empathy* was assessed through two tasks targeting its cognitive and affective components, inspired by the Multifaceted Empathy Task [22]:

- In *the Cognitive Empathy task (CEt),* participants are shown images depicting individuals in emotionally salient or neutral situations and are asked to identify how the protagonist is feeling.
- The *Affective Empathy task (AEt)* follows the same procedure but requires participants to indicate their own emotional response to the observed situation.

Both tasks include 3 trials each (score range: 0–3), with responses provided using a three-point categorical scale, and scored as correct or incorrect. 

*Social norms understanding* was assessed through*:*

*• The Social Norms Understanding task (SNUt),* which evaluates knowledge of moral and social rules. Its content is partially inspired by the Social Norms Questionnaire [23], but visual stimuli were used to enhance ecological validity. Participants were shown photographs depicting socially acceptable or unacceptable behaviors and were asked to judge whether the behavior was morally acceptable or not. A brief verbal explanation was required to ensure the correct interpretation of the situation. It includes 6 trials (score range: 0–6), scored as correct or incorrect.

The BE-Social can be administered in approximately 15 min. Each task yields a task-specific score and contributes to a subscale score, and a total score is computed by summing performance across all tasks.

### Procedure

All participants completed the BE-Social and the MoCA [17] and underwent an individual assessment in a quiet clinical or laboratory setting. Before testing, demographic information and relevant medical history were collected through a semi-structured interview. A subgroup of healthy controls (n = 82) additionally completed an extended assessment to examine the convergent and divergent validity of the BE-Social. Convergent validity was assessed using established performance-based measures of social cognition targeting emotion recognition (Ekman 60 Faces Test; EK-60) [24], Theory of Mind (Story-based Empathy Task; SET) [20], complex affective mental state inference (Reading the Mind in the Eyes Test; RMET) [25], dispositional empathy (Interpersonal Reactivity Index; IRI) [25], and social norm understanding (interpersonal and intrapersonal subscales of the Edinburgh Social Cognition Test) [26]. Divergent validity was examined using non-social cognitive measures assessing executive functions (Frontal Assessment Battery; FAB) [27], verbal short-term and working memory (Digit Span Forward and Backward) [28], as well as cognitive flexibility (Trail Making Test B/A ratio) [29]. Another subgroup of healthy controls (n = 30) participated in the test–retest reliability assessment, with a second administration of the BE-Social occurring 30–40 days after the first assessment.

Patients were administered the BE-Social together with a neuropsychological battery to characterize their socio-cognitive functioning and to examine associations with established measures of social cognition.

Social cognition was assessed using the EK-60 [24], the SET [20], the IRI [25] and the Social Norm Questionnaire (SNQ; [26]), selected to cover the main social cognition domains targeted by the BE-Social. Global cognitive functioning and executive functions were assessed using the MoCA [17], and the FAB [27], respectively.

Based on their performance on social perception (assessed *via* EK-60), ToM (assessed *via* SET), empathy (assessed *via* IRI), and social norms understanding (assessed *via* SNQ), patients were classified as defective (DEF) or non-defective (NON-DEF) with respect to social cognition. Specifically, social cognition impairment was defined as the presence of either at least a defective (below cut-off) performance (Equivalent Score, ES = 0) in one of the assessed social cognition domains or two borderline performances (ES = 1) across two different tests. Because Italian normative data for the SNQ are currently unavailable, a cut-off score of ≤ 15 was derived from the Italian healthy control sample reported by Isernia et al. [26] following the conventional procedure [30].

The overall assessment session lasted approximately 45–60 min, depending on the participant group and the number of measures administered. All assessments were carried out by licensed neuropsychologists or neuropsychology trainees who had received specific training on test administration and scoring procedures.

### Statistical analyses

Exploratory analyses were conducted to examine the internal structure of the BE-Social and the association of each task with the composite (total) score (see Supplementary Materials S2). Given the screening purpose of the instrument, these analyses were also intended to evaluate the appropriateness of computing a total score.

Internal consistency was evaluated using McDonald’s omega total, computed from a polychoric correlation matrix, as recommended for ordinal and skewed data [31].

*Test–retest reliability* was assessed using intraclass correlation coefficients (ICCs), the Wilcoxon signed-rank test, and Spearman correlations. *Convergent and divergent validity* were examined using Spearman’s rank correlation coefficients, as most measures did not meet the assumptions required for linear modeling. To control for multiple comparisons, the significance threshold was adjusted using the Bonferroni correction. Raw p-values are reported, and significance was determined based on the adjusted alpha.

To explore the effect of demographic variables (age, sex and education), a regression-based approach was adopted, considering age and education as continuous variables. To derive *normative thresholds* and adjust raw BE-Social scores for the significant demographic predictors the Equivalent Score (ES) method [32, 33] was applied. 

To address known limitations of this approach, particularly the selection of the “best transformation” based solely on R2, a model selection procedure proposed by Arcara (2024) [34] was also applied. This procedure systematically explores different transformations and their combinations for each predictor, selecting the optimal model based on the Akaike Information Criterion (AIC), aimed at balancing model fit and complexity. The models identified by the two approaches were compared to select the most appropriate solution. Subsequently, the conventional ES computation procedure was applied [32]. To explore *clinical usability*, receiver operating characteristic (ROC) analyses were performed using the demographically adjusted BE-Social total score, comparing (1) the entire clinical sample with the normative sample and (2) DEF versus NON-DEF patients.

Before these analyses, preliminary comparisons were conducted to verify that BE-Social performance was not primarily driven by diagnostic category but rather by the presence of social cognition impairment. Specifically neurological and psychiatric patients were compared on global cognition (MoCA), executive functioning (FAB), and BE-Social total score. Additional analyses compared DEF and NON-DEF patients within each clinical group, as well as neurological and psychiatric patients classified as DEF. No significant differences were found between neurological and psychiatric patients in MoCA, FAB or BE-Social performance, either in the overall sample or among impaired individuals only (i.e., DEF), suggesting a comparable socio-cognitive profile across diagnostic categories. Therefore, neurological and psychiatric patients were combined in the subsequent ROC analyses. Detailed results of these preliminary comparisons are reported in the Supplementary Materials S3.

Finally, associations between BE-Social performance and socio-cognitive measures were examined using Spearman’s rank correlations. To account for individual differences in general cognitive functioning, partial correlations controlling for MoCA were computed, with Bonferroni correction applied for multiple comparisons.

All analyses were conducted using R version 4.4.3 (R Core Team, 2025) and Jamovi version 2.3.28 (The Jamovi Project, 2022).

## Results

Demographic characteristics of the normative sample are summarized in Table 2.

**Table 2:** Demographic and cognitive data of the normative sample

*N* = 466 (282 females)	Mean raw score ± SD (min–max)
**Age**	50.25 ± 19.42 (20–93)
**Education**	13.57 ± 3.96 (5–23)
**Montreal Cognitive Assessment (MoCA)**	26.46 ± 2.95 (14–30)
**Brief Examination of Social Abilities (BE-Social)**	27.42 ± 2.64 (16–30)
**Frontal Assessment Battery (FAB)**^**a**^	17.50 ± 0.85 (14–18)
**Reading the Mind in the Eyes (RMET)**^**a**^	26.91 ± 3.48 (10–34)
**Ekman 60 Faces test (EK-60)**^**a**^	50.59 ± 4.44 (35–58)
**Story Based Empathy task (SET)**^**a**^	17.50 ± 0.84 (14–18)
**Interpersonal Reactivity Index (IRI)**^**a**^	71.60 ± 9.88 (35–93)
**Edinburgh Social Cognition Test (ESCoT)**	
**ESCoT intrapersonal subscale**^**a**^	27.23 ± 2.43 (18–30)
**ESCoT interpersonal subscale**^**a**^	25.98 ± 3.12 (18–30)
**Digit Span Forward (DSF)**^**a**^	6.34 ± 0.91 (4–9)
**Digit Span Backward (DSB)**^**a**^	5.33 ± 1.12 (3–8)
**Trail Making Test B/A (TMT B/A)**^**a**^	2.89 ± 0.98 (1.50–6.94)

^a^Data available for a subsample of *N* = 82 participants; *SD* standard deviation

Exploratory analyses indicated that all tasks were positively and consistently associated with overall performance. Factorial analyses revealed the presence of a dominant first factor, with all tasks contributing consistently to the overall construct, and without clear evidence of a hierarchical structure, supporting the plausibility of computing a total score. Detailed results are reported in the Supplementary Materials S2.

Internal consistency of the BE-Social total score was satisfactory, with McDonald’s omega equal to 0.83. Test–retest reliability showed acceptable temporal stability. Mean total scores at baseline (M = 27.62 ± 2.28) and at retest (M = 28.03 ± 2.01) did not differ significantly (W = 88.5, p = 0.221). Performance across sessions was moderately and significantly correlated (rho = 0.62, p < 0.001). In addition, the intraclass correlation coefficient confirmed moderate test–retest reliability (ICC = 0.66, 95% CI [0.41, 0.82]).

Convergent validity analyses showed that, considering the adjusted alpha level (α = 0.008), the BE-Social total score was significantly associated with performance on the EK-60 and the SET, whereas no significant associations emerged with the RMET, IRI, or ESCoT social norms subscales under the Bonferroni-adjusted alpha threshold (Table 3). Regarding divergent validity, applying the adjusted significance level (α = 0.01), the BE-Social total score was not significantly associated with executive functions, verbal memory, working memory, and attentional-shifting measures, while a significant association emerged with the MoCA (Table 3). Both the traditional [33] and AIC-based procedure [34] identified age and education as significant predictors, whereas sex did not contribute significantly. The AIC-based procedure selected a model including log-transformed age and square-root transformed education, which showed marginally better fit indices compared to the model identified by the traditional approach and was therefore retained as the final model. The final regression was statistically significant (F_(2, 463)_ = 70.24, p < 0.001; adjusted R2 = 0.230), with lower BE-Social performance associated with increasing age and lower education. Detailed regression coefficients, adjustment equation, tolerance limits, and Equivalent Score thresholds are reported in Table 4.

**Table 3:** Correlations between the BE-Social total score and other cognitive measures in healthy controls

Social cognition tests		Other cognitive tests	
**EK-60**	***rho* = 0.34; *p* = 0.001**		
**RMET**	*rho* = 0.26; *p* = 0.016	**MoCA**	***rho* = 0.34; *p* = 0.001**
**SET**	***rho* = 0.30; *p* = 0.006**	**FAB**	*rho* = 0.13; *p* = 0.268
**IRI**	*rho* = 0.11; *p* = 0.314	**DSF**	*rho* = −0.01; *p* = 0.872
**ESCoT interpersonal**	*rho* = 0.06; *p* = 0.581	**DSB**	*rho* = 0.02; *p* = 0.812
**ESCoT intrapersonal**	*rho* = 0.01; *p* = 0.872	**TMT B/A**	*rho* = −0.02; *p* = 0.846

Spearman’s rank correlations. Bonferroni correction was applied for multiple comparisons (adjusted α = .008 for social cognition measures; α = .01 for other cognitive measures). Significant correlations after correction are reported in bold. For acronymous, see Table 2

**Table 4:** Equivalent Score thresholds for the BE-Social total score and demographic adjustment equation

oTL	iTL	ES = 0	ES = 1	ES = 2	ES = 3	ES = 4
21.99	23.14	≤ 21.99	22.00–24.01	24.02–26.40	26.41–28.08	≥ 28.09
AS = RS—2.0319783 × [ln100(age)—3.810724]—0.8415957 × [√(education)—3.639042]						

*ES* Equivalent Score, *AS* Adjusted Score, *RS* Raw Score, *oTL* outer tolerance limit, *iTL* inner tolerance limit

**Fig. 1:**
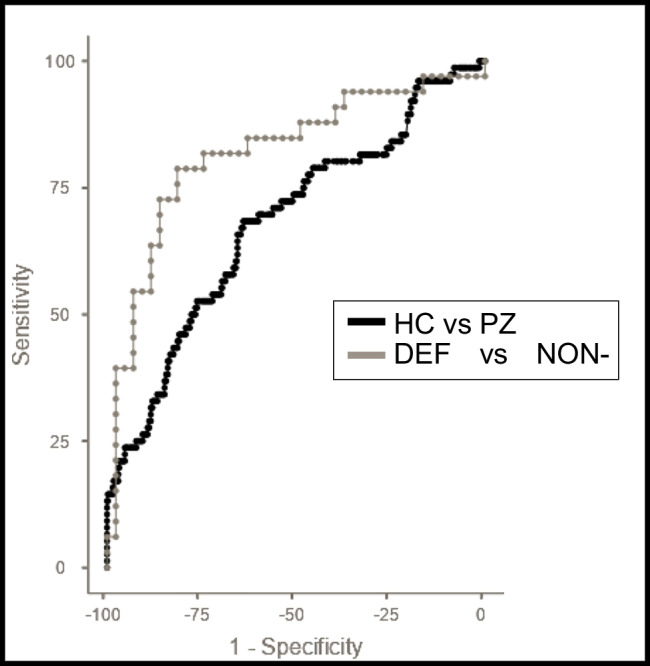
Receiver operating characteristic (ROC) curves for the BE-Social total score The figure illustrates the discriminative performance of the BE-Social in differentiating healthy controls (HC) from patients (PZ) and in distinguishing impaired in social cognition (DEF) from non-impaired (NON-DEF) patients

Among the 76 patients included in the study, 33 (43%) were classified as defective with respect to social cognition, with similar proportions across neurological (approximately 45%) and psychiatric (approximately 41%) subgroups. Preliminary analyses showed that BE-Social performance did not differ significantly between neurological and psychiatric patients, nor between DEF neurological and DEF psychiatric patients. Detailed results are reported in the Supplementary Materials S3. ROC analyses (Fig. 1) showed that the BE-Social adjusted total score had a moderate discriminative accuracy in distinguishing patients from HC (AUC = 0.68), and a good discriminative accuracy in distinguishing DEF from NON-DEF patients (AUC = 0.82). Partial Spearman’s correlations (controlling for MoCA) demonstrated that the BE-Social total score was significantly associated with EK-60, SET, and SNQ after Bonferroni correction, whereas no significant association emerged with IRI. Detailed correlation coefficients are reported in Table 5.

**Table 5:** Partial correlations between BE-Social and cognitive/socio-cognitive measures in patients

**EK-60**	***rho* = 0.47; *p* < 0.001**
**SET**	***rho* = 0.28; *p* = 0.014**
**SNQ**	***rho* = 0.33; *p* = 0.004**
**IRI**	*rho* = 0.19; *p* = 0.107
**FAB**	*rho* = 0.22; *p* = 0.053

Partial correlations controlling for MoCA. Bonferroni correction was applied for multiple comparisons (adjusted α = .01). Significant correlations after correction are reported in bold

## Discussion

The present work develops and validates a novel screening test for assessing social cognition, providing Italian normative data and evidence on its clinical usability across diverse clinical conditions.

Overall, the BE-Social demonstrates satisfactory internal consistency, with McDonald’s omega exceeding the commonly accepted threshold for internal reliability [35].

Although the battery was designed to cover multiple theoretically distinct domains (i.e., social perception, ToM, empathy, and social norm understanding), the use of a global index of socio-cognitive efficiency (total score) appears more appropriate in light of the observed structure, which was best represented by a single dominant factor, and the primary aim of the instrument as a clinical screener. This choice enhances the feasibility of the BE-Social as screening in routine clinical settings, where time constraints often limit the use of more extensive batteries. At the same time, it allows clinicians to formulate qualitative hypotheses about domain-specific difficulties that may be further explored using second-level assessment. Test–retest reliability is moderate (ICC = 0.67), indicating that BE-Social scores remain relatively stable over time [36].

Convergent and divergent validity analyses support the construct validity of the BE-Social. The total score shows significant associations with established performance-based measures of emotion recognition and ToM (i.e., EK-60 and SET, respectively), confirming that the battery captures core socio-cognitive processes. By contrast, there are no significant associations with self-report measures of empathy (i.e., IRI) or with tasks assessing more complex or metacognitive aspects of social cognition, such as the RMET or ESCoT. These discrepancies may reflect methodological and/or conceptual differences between instruments. Specifically, the RMET assesses complex emotion recognition involving the inference of subtle affective states, whereas the BE-Social primarily focuses on basic emotions identification. Similarly, the BE-Social Empathy tasks require participants to evaluate others and their own emotional state in visually presented situations using a simple three-point valence scale (positive, negative, neutral), without the fine-grained differentiation of affective experience captured by the IRI. The absence of association with the ESCoT subscales may also result from similar differences. In the BE-Social, social norms understanding is evaluated by presenting explicit moral violations and requiring a binary (yes/no) judgment, whereas the ESCoT involves open-ended responses and metacognitive reasoning about socially ambiguous situations.

Importantly, the absence of significant associations with executive functions, attention, and verbal memory supports the specificity of the BE-Social for socio-cognitive functioning. However, the modest association observed with global cognitive functioning suggests that, although cognitive load was minimized, socio-cognitive performance still depends partly on general cognitive efficiency. This is not unexpected: as several authors have argued, intact cognitive functioning is necessary, even if not sufficient, for the expression of social cognition [6, 37, 38]. Multiple cognitive resources operate in parallel with socio-cognitive skills, but the dynamic of their interaction remains debated [6, 37]. Accordingly, the correlation with global cognition should not be interpreted as a lack of specificity, but rather as evidence that social-cognitive performance may emerge from the coordinated engagement of domain-general and domain-specific mechanisms.

Regarding demographic influences, lower BE-Social scores are associated with increasing age and lower educational attainment, consistent with previous evidence showing age-related decline in social-cognitive abilities, particularly in social perception and ToM [39, 40], as well as evidence of poorer social cognitive functioning associated with lower levels of education, as demonstrated by previous normative studies of social cognition tests [20, 24]

In the clinical sample, a substantial proportion of patients (43%) show impaired socio-cognitive performance, despite being recruited independently of social cognition complaints. This highlights the clinical relevance of systematic socio-cognitive screening. Notably, the prevalence of impairment is comparable across neurological and psychiatric groups, supporting the conceptualization of social cognition dysfunctions as a transdiagnostic feature, rather than a disorder-specific phenomenon [2–4, 8]. ROC analyses demonstrate good accuracy in discriminating defective from non-defective patients, whereas discrimination between patients and HC is weak. These findings indicated that the BE-Social is effective in identifying social cognition impairment within clinical populations, but not in discriminating patients from healthy individuals per se. Correlational analyses in the clinical sample further support the construct validity of the BE-Social, as it was significantly associated with measures of emotion recognition, ToM, and social norm understanding. Importantly, these associations remain significant after controlling for the level of global cognitive functioning (i.e., MoCA scores), suggesting that BE-Social captures a level of socio-cognitive abilities that cannot be fully accounted for by general cognitive efficiency. The lack of association with self-reported empathy, also observed in the HC sample, is consistent with the notion that performance-based assessments and self-report measures may tap partially distinct levels of socio-cognitive functioning. Overall, these findings support the use of the BE-Social total score as a reliable and clinically meaningful screening index of social cognition in neurological and psychiatric populations.

### Limitations and future directions

A moderate ceiling effect was observed in the normative sample, suggesting that the BE-Social may be less sensitive to subtle individual differences among individuals with high socio-cognitive performance. Moreover, although the clinical sample was intentionally heterogeneous to reflect real-world practice, the limited size of specific diagnostic subgroups prevents further disorder-specific analyses. In addition, the classification of socio-cognitive impairment relies on performance-based neuropsychological measures in the absence of a gold standard or direct indices of real-world social functioning. Future studies should therefore examine the relationship between BE-Social performance and ecological or caregiver-reported outcomes to further establish its clinical relevance. Future developments should also consider formal cross-cultural adaptation and validation procedures to investigate the applicability of the BE-Social across different sociocultural contexts.

## Conclusions

The BE-Social represents a promising instrument for a rapid screening of social cognition in clinical settings. By providing a brief and standardized multidomain assessment, it may facilitate the systematic integration of social cognition evaluation into routine neuropsychological practice, supporting a more comprehensive clinical characterization.

The BE-Social materials, administration instructions, and scoring sheet are freely available on the Open Science Framework (OSF). (https://osf.io/2w5hn/: [https://osf.io/2w5hn/overview?view_only=252d2c1a8511402ab6e6ae07166c1c3f]).

## Supplementary Information

Below is the link to the electronic supplementary material.

Supplementary file1 (DOCX 99 KB)

## Data Availability

Datasets associated with the present study can be made available on Zenodo (https://zenodo.org/records/18978754) upon reasonable request to the Corresponding Author (Dr. Maria Franca, m.franca@campus.unimib.it).
