# Supplementary material for: Normative data and clinical usability of the brief examination of social abilities: a novel screening test for social cognition
Source: Neurol Sci. 2026 Jun 15;47(7):567. doi: 10.1007/s10072-026-09173-3 (PMC13265596; doi:10.1007/s10072-026-09173-3)
Supplement: Supplementary file 1 — Supplementary file1 (DOCX 99 KB) [file 10072_2026_9173_MOESM1_ESM.docx]

**Supplementary Materials**

**Supplementary 1 (S1). Pilot study and development of the BE-Social**

**S1. Participants**

Thirty-two healthy volunteers participated in the pilot study (11 males). Participants were recruited through the experimenters’ personal networks and word of mouth. Age ranged from 20 to 82 years (M = 44.28, SD = 18.08), and years of education ranged from 5 to 20 (M = 15.34, SD = 3.53).

**S1. Procedure and materials**

The pilot study was conducted online using the Qualtrics platform. Each BE-Social task was implemented as a separate block, and the order of the blocks was randomized across participants. Within each block, both trials and response options were presented in randomized order. The following tasks were administered:

- *Identity Recognition task*: Four trials were presented. Each trial consisted of a target face and three alternative faces, from which participants were required to select the correct match. The target face was displayed at the top of the screen, outlined in red and shown from a frontal view, while the three alternatives were presented below from different angles and under different lighting conditions. Trials were created using photographs from the FEI face database [1] with the author’s permission and converted into black and white.
- *Emotion Recognition task:* A total of 72 photographs were presented, depicting six actors (three males and three females) expressing six basic emotions (anger, disgust, sadness, surprise, happiness, and fear). Each emotional expression was presented against both a white and a black background (36 stimuli per background). New stimuli were created to ensure originality and to avoid overlap with existing second-level tests. Actors were trained to reproduce emotional expressions according to Ekman’s Facial Action Coding System (FACS). Participants selected the corresponding emotion label from six alternatives and subsequently rated the perceived intensity of the emotion on an 8-point Likert scale “How intense was the emotion you perceived?”. At the end of the task, participants were asked to indicate their preferred background color.
- *Story Inference task:* Nine sequential photographic vignettes were presented (three per condition: causal inference, emotion inference, and intention inference). All scenarios were created ad hoc for this study. After observing each vignette, participants selected the most appropriate ending among three alternatives.
- *Mini Faux Pas task:* Four short written stories were administered. Stories were adapted from the Italian translation of the Faux Pas Test for adults (unpublished adaptation by Massaro, Liverta Sempio, & Marchetti) with simplified narratives and a modified scoring method. After each story, participants answered two control questions assessing story comprehension and faux pas detection, followed by Theory of Mind (ToM) questions. The ToM section comprised three questions assessing: (i) understanding of why the statement constituted a faux pas, (ii) inference of the speaker’s intentions, and (iii) inference of the emotional impact on the recipient. Responses were provided in a multiple-choice format with three alternatives. For each ToM question, one option was fully correct, one was partially correct, and one was incorrect, allowing a more fine-grained assessment of second-order ToM.
- *Empathy tasks (Cognitive and Affective Empathy)*: Fourteen images depicting protagonists in emotionally salient (positive or negative) or neutral situations were initially presented. 11 images were obtained from the free database Pixabay.com, and 3 were created ad hoc. For each image, participants indicated (i) how the protagonist was feeling (Cognitive Empathy) and (ii) how they themselves felt when viewing the image (Affective Empathy), using a three-point visual scale. Participants also rated emotional arousal on an 8-point scale. Stimuli included four positive, two neutral, and eight negative scenes.
- *Social Norms Understanding task*: Eight photorealistic images depicting socially acceptable or unacceptable behaviors (four moral and four immoral actions) were presented. Images were selected from Pixabay.com and created ad hoc. Participants judged whether the behavior was morally acceptable (yes/no) and provided a brief verbal explanation to ensure correct interpretation of the rule involved.

The overall testing session lasted approximately one hour. At the end of the session, participants were asked whether any task was unclear and were invited to provide qualitative feedback and suggestions for improvement.

**S1. Results**

Descriptive analyses were conducted to evaluate task feasibility, stimulus recognizability, and response distributions. Decisions regarding item retention were based on accuracy rates, response distributions, arousal and intensity ratings (where applicable), and qualitative feedback.

- *Identity Recognition task: All* items were recognized with accuracy rates above 90%. One item in which a distractor differed in gender from the target was excluded, as it was considered overly easy and potentially less informative. Three trials were retained.
- *Emotion Recognition task:* Approximately 66% of participants reported a preference for the white background, which was also associated with higher accuracy rates for most emotions (except for fear). Six trials were selected (one per emotion), ensuring that each actor contributed one stimulus. Fear was the most difficult emotion to recognize; therefore, the trial with the highest accuracy (78%) was selected. For the remaining emotions, trials with accuracy above 80% and mean intensity ratings ≥ 5 were retained.
- *Story Inference task:* Eight out of nine vignettes showed accuracy rates above 80%. One emotion inference story performed at chance level (50%) and was excluded. Three final stories (one per condition) were selected, prioritizing those with higher accuracy and fewer distracting elements.
- *Mini Faux Pas task:* Of the four stories, one was excluded because only 22% of participants correctly identified the faux pas. In another story, although faux pas detection was high (87.5% accuracy on control questions), performance on subsequent ToM questions was poor, ranging from 26 to 37%. The two remaining stories were retained, as faux pas detection exceeded 70% and accuracy on ToM questions ranged between approximately 74% and 91%.
- *Empathy tasks:* Participants generally showed higher accuracy on Cognitive Empathy than on Affective Empathy items. For the Affective Empathy task, neutral stimuli were excluded, as they are not expected to reliably elicit an affective empathic response. Selection prioritized stimuli with high arousal ratings (≥ 6) and accuracy ≥ 80%, resulting in one positive and two negative items. For the Cognitive Empathy task, neutral stimuli were retained as control items assessing recognition of emotional absence. One positive, one neutral, and one negative item were selected, prioritizing clarity and accuracy.
- *Social Norms Understanding task:* All eight stimuli were recognized with accuracy above 90%. To reduce task length, all four immoral stimuli and the two least ambiguous moral stimuli were retained, for a total of six trials.

**S1. Conclusion**

The pilot study confirmed the feasibility and clarity of the BE-Social tasks and the suitability of the newly developed stimuli. Items that were poorly recognized, ambiguous, or excessively easy were excluded, resulting in a concise and interpretable screening test. The pilot phase also allowed optimization of task length, ensuring that the final instrument remains suitable for bedside and routine clinical assessment. Overall, these findings supported the design choices underlying the BE-Social and provided a solid foundation for subsequent validation and clinical usability analyses. Based on these results, the final version of the BE-Social includes seven tasks with a reduced and optimized number of trials. The final structure, including domains, number of trials per task, and scoring ranges, is reported in Table S1.

| **Table S1**. Structure and scoring of the BE-Social | | | |
| --- | --- | --- | --- |
| **Domain** | **Task** | **N° of trials** | **Score range** |
| **Social Perception** | *Identity Recognition task* | 3 | 0–3 |
|  | *Emotion Recognition task* | 6 | 0–6 |
| **Theory of Mind** | *Story Inference task* | 3 | 0–3 |
|  | *Mini Faux Pas task* | 2 | 0–6 |
| **Empathy** | *Cognitive Empathy task* | 3 | 0–3 |
|  | *Affective Empathy task* | 3 | 0–3 |
| **Social Norms comprehension** | *Social Norms Understanding task* | 6 | 0–6 |
| **Total BE-Social** | -- | -- | **0–30** |

**Supplementary 2 (S2). Internal structure of the BE-Social**

**S2. Methods**

To examine the internal structure of the BE-Social, parallel analysis and inspection of the scree plot were conducted on the polychoric correlation matrix to guide factor retention. Exploratory factor analysis (EFA) was then performed using maximum likelihood extraction to estimate the latent factor structure underlying the seven tasks. Data suitability was assessed using the Kaiser–Meyer–Olkin (KMO) measure of sampling adequacy and Bartlett’s test of sphericity.

Spearman’s rank correlation coefficients were calculated between each task score (Identity Recognition task, Emotion Recognition task, Story Inference task, Mini Faux Pas task, Cognitive Empathy task, Affective Empathy task, and Social Norm Understanding task) and the BE-Social total score to quantify the association of each task with overall performance. Spearman’s correlation was selected because subtask scores were discrete and showed marked negative skewness. These analyses provide a descriptive index of how strongly each task is associated with the overall composite score.

In addition, corrected item (task) – total correlations (i.e., the correlations between each task score and the total score computed excluding that task) were calculated based on the polychoric correlation matrix. This analysis was performed to estimate the degree to which each task is aligned with the common construct represented by the composite score.

**S2. Results**

Regarding the internal structure of the BE-Social, the KMO measure of sampling adequacy was .75, indicating adequate suitability for factor analysis. Subtest-specific KMO values ranged from .68 to .83. Bartlett’s test of sphericity was statistically significant, χ²(21) = 708.26, *p* < .001, indicating that the correlation matrix was suitable for factor analysis.

Parallel analysis suggested the presence of four factors based on common factor extraction, whereas principal component analysis indicated a single dominant component (Figure S2.1). Inspection of the scree plot and the relative magnitude of the first eigenvalue supported the presence of a strong general factor.

The one-factor EFA solution yielded positive loadings for all subtests (.39–.81; Table S2.1). The general factor explained 32% of the variance and showed acceptable model fit (RMSR = .067). These findings support the plausibility of an essentially unidimensional structure and the use of a total score.

Spearman’s rank correlations between each BE-Social task and the total score were all positive and statistically significant, ranging from rho = .27 to rho = .80 (all *p* <.001; Table S2.2). These results indicate moderate to strong associations between individual task performances and overall BE-Social performance.

Corrected item–total correlations ranged from .33 to .64 (Table S2.2). All tasks showed adequate internal consistency with the composite score, indicating that each task was coherently associated with the underlying construct represented by the BE-Social total score.

**
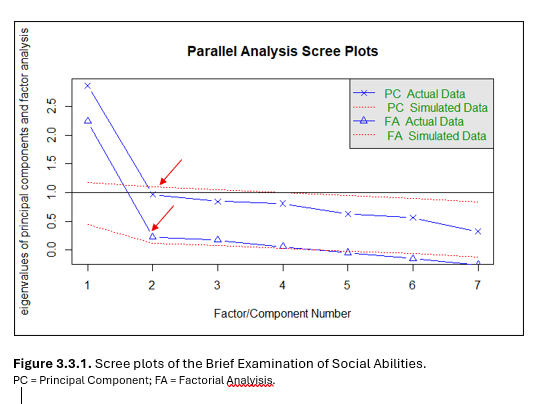
Figure S2.1.** Parallel analysis scree plot for the BE-Social.

The plot compares observed eigenvalues from factor analysis (FA) and principal component analysis (PC) with eigenvalues obtained from randomly simulated data. Inspection of the scree plot indicates a dominant first factor/component, with only the first observed eigenvalue clearly exceeding the corresponding simulated eigenvalue.

| **Table S2.1.** One-factor exploratory factor analysis for BE-Social. | |
| --- | --- |
| **Task** | **Loading** |
| **Identity Recognition task** | 0.403 |
| **Emotion Recognition task** | 0.563 |
| **Story Inference task** | 0.664 |
| **Mini Faux Pas task** | 0.441 |
| **Cognitive Empathy task** | 0.556 |
| **Affective Empathy task** | 0.388 |
| **Social Norm Understanding task** | 0.813 |
| Loadings are based on maximum likelihood extraction from the polychoric correlation matrix (no rotation). | |

| **Table S2.2.** Associations between each BE-Social task and the total score (Spearman’s rho) and corrected item–total correlations (r.drop) | | |
| --- | --- | --- |
| **Task** | **Correlation with total BE-Social score (rho)** | **Item-total correlation** |
| **Identity Recognition task** | .38^a^ | .38 |
| **Emotion Recognition task** | .60^a^ | .53 |
| **Story Inference task** | .36^a^ | .52 |
| **Mini Faux Pas task** | .80^a^ | .42 |
| **Cognitive Empathy task** | .35^a^ | .46 |
| **Affective Empathy task** | .33^a^ | .33 |
| **Social Norm Understanding task** | .27^a^ | .64 |
| ^a^ = All correlations were significant at p < .001 and remained significant after Bonferroni correction for multiple comparisons. | | |

**Supplementary 3 (S3). Preliminary clinical group comparisons**

**S3. Methods**

To justify the combination of neurological and psychiatric patients in subsequent analyses and to support the transdiagnostic application of the instrument, group comparisons were performed between: (i) Neurological versus psychiatric patients; (ii) Defective (DEF) versus non-defective (NON-DEF) patients within each clinical group; (iii) Neurological DEF versus psychiatric DEF patients. Depending on variable distribution and measurement level, group differences were tested using independent-samples t tests or Mann–Whitney U tests for continuous variables, and χ² tests for categorical variables. BE-Social total score, global cognitive functioning (MoCA), executive functions (FAB), and standardized social cognition measures (EK-60, SET, IRI, SNQ) were considered. The significance level was set at α = .005 (.05/9) in comparison between NEURO and PSYCH (Table S3.1) and α = .01 (.05/5) in Table S3.2.

**S3. Results**

*(i) Neurological vs psychiatric patients.*

No significant differences were observed between the two clinical groups in social cognition measures, including the BE-Social total score, nor in non-social cognitive measures (NEURO vs. PSYCH; Table S3.1).

*(ii) Defective vs. non-defective patients within clinical subgroups.*

Among the 76 patients included in the study, 33 (43%) were classified as defective (DEF) with respect to social cognition, based on performance on the EK-60, SET, IRI, and SNQ. Classification criteria are described in the Methods section. Within the defective sample, the most frequently impaired performances were at EK-60 and SNQ, whereas deficits in SET and IRI were less frequent (Figure S3.1).

In the *neurological* group, DEF patients showed significantly lower global cognitive functioning than NON-DEF patients, as measured by the MoCA (*p* = .001), as well as significantly lower performance on the BE-Social total score (*p* = .001; Table S3.2). No significant differences were observed between DEF and NON-DEF neurological patients with respect to age, years of education, or sex distribution (χ²(1) = 0.12, *p* = .734), nor in executive functioning as assessed by the FAB (*p* = .132).

Similarly, in the *psychiatric* group, DEF patients demonstrated significantly lower performance than NON-DEF patients on the MoCA (*p* < .001) and on the BE-Social total score (p < .001; Table S3.2). No significant differences were found between DEF and NON-DEF psychiatric patients with respect to demographic variables (sex: χ²(1) = 1.09, *p* = .295). In contrast to the neurological group, DEF psychiatric patients also showed significantly lower FAB scores compared to NON-DEF patients (*p* = .001).

*(iii) Defective neurological vs defective psychiatric patients.*

Direct comparisons between DEF neurological and DEF psychiatric patients revealed no significant differences in demographic variables, global cognitive functioning, or BE-Social total score, indicating comparable levels of social cognition impairment across clinical categories.

| **Table S3.1.** Comparison between neurologic and psychiatric groups | | | | |
| --- | --- | --- | --- | --- |
|  | **NEURO**  (n = 42; 20 F) | **PSYCH**  (n = 34; 17 F) | **Statistics** | **p** |
| **Age** | 60.21 ± 16.15 | 52.53 ± 9.23 | *t*_74_ = 2.47 | .016 |
| **Education** (years) | 13.26 ± 3.79 | 11.94 ± 3.63 | U = 860 | .118^a^ |
| **MoCA** | 21.98 ± 4.25 | 20.03 ± 4.37 | U = 893.5 | .060^a^ |
| **FAB** | 14.43 ± 3.22 | 14.76 ± 3.17 | U = 666 | .616^a^ |
| **EK-60** | 41.40 ± 8 | 41.15 ± 10.40 | t_74_ = 0.12 | .903 |
| **SET** | 14.43 ± 3.43 | 14.41 ± 2.83 | U = 753 | .685^a^ |
| **IRI** | 62.57 ± 14.10 | 65.18 ± 14.20 | t_74_ = -0.80 | .427 |
| **SNQ** | 17.52 ± 2.46 | 17.50 ± 1.88 | t_74_ = 0.05 | .963 |
| **BE-Social** | 25.46± 3.74 | 24.26±4.14 | U= 842.5 | .180^a^ |
| Student’s t test was used for mean comparisons; ^a^ = Mann-Whitney U test was applied; MoCa = Montreal Cognitive Assessment; FAB = Frontal Assessment Battery; EK-60 = Ekman 60 Faces Test; SET = Story-based empathy task; IRI = Interpersonal Reactivity Index; SNQ = Social Norm Questionnaire; | | | | |

**Figure S3.1. Socio-cognitive profile of defective patients.**
Distribution of performances below cut-off (Equivalent Score = 0), borderline (ES = 1), and within normal range (ES > 1) across emotion recognition (EK-60), theory of mind (SET), empathy (IRI), and social norms understanding (SNQ) in patients classified as defective (DEF).

| **Table S3.2.** Demographics and test scores of defective and non-defective patients in the neurological and psychiatric subgroups | | | | | | | | | |
| --- | --- | --- | --- | --- | --- | --- | --- | --- | --- |
|  | **NEURO (n = 42)** | | | | **PSYCH (n = 34)** | | | | **Def NEURO vs PSYCH** |
|  | **Def**  (n = 19; 8 F) | **Non-def**  (n = 23; 12 F) |  | **p** | **Def**  (n = 14; 5 F) | **Non-def**  (n = 20;12 F) |  | **p** | **p** |
| **Age** | 63.42±18.61 | 57.57±13.65 | *U*=159 | .135^a^ | 54.29±8.05 | 51.30±9.98 | *t_32_*= -.92 | .361 | .096^#^ |
| **Education** | 12.84±4.37 | 13.61±3.29 | *U*=231 | .756^a^ | 11.29±2.92 | 12.40±4.06 | *t_32_*= .87 | .386 | .214^a,#^ |
| **MoCA** | 19.68±4.11 | 23.87±3.39 | *U*=344.5 | **.001^a^** | 17.14±4.94 | 22.05±2.44 | *t_32_*= 3.83 | **<.001** | .158^a,#^ |
| **FAB** | 13.53±3.67 | 15.17±2.64 | *U*=278 | .132^a^ | 12.57±3.76 | 16.30±1.34 | *U* = 230 | **.001^a^** | .470^#^ |
| **BE-Social** | 23.55±3.88 | 27.04±2.82 | *U*=344.5 | **.001^a^** | 20.89±3.83 | 26.62±2.35 | *t_32_*= 5.40 | **<.001** | .059^#^ |
| Student’s *t* test was used for mean comparisons; ^a^ = Mann-Whitney *U* test was applied; ^#^ = p values in the last column refer to comparisons between defective neurological and defective psychiatric patients | | | | | | | | | |

[1] Thomaz, C. E. (2012). *FEI face database.* FEI Face Database, 11, 46–57.
